# CIViC MCP: Integrating Large Language Models with the Clinical Interpretations of Variants in Cancer

**DOI:** 10.1101/2025.10.13.682185

**Published:** 2025-10-16

**Authors:** Lars Schimmelpfennig, Quentin Cody, Joshua McMichael, Adam C. Coffman, Jason Saliba, Arpad Danos, Susanna Kiwala, Alex Wagner, Javier Sanz-Cruzado, Jake Lever, Malachi Griffith, Obi L. Griffith

**Affiliations:** 1 Department of Medicine, Washington University School of Medicine, St. Louis, Missouri, USA; 2 Department of Genetics, Washington University School of Medicine, St. Louis, Missouri, USA; 3 Independent Researcher, Philadelphia, PA, USA; 4 McDonnell Genome Institute, Washington University School of Medicine, St. Louis, Missouri, USA.; 5 Steve and Cindy Rasmussen Institute for Genomic Medicine, Nationwide Children’s Hospital, Columbus, Ohio, USA; 6 Department of Pediatrics, The Ohio State University College of Medicine, Columbus, Ohio, USA; 7 Information Retrieval Group, School of Computing Science, University of Glasgow; 8 AI4BioMed Lab, School of Computing Science, University of Glasgow; 9 Siteman Cancer Center, Washington University School of Medicine, St. Louis, Missouri, USA.

## Abstract

**Summary:**

The Clinical Interpretation of Variants in Cancer (CIViC) knowledgebase provides a community-driven, open-source platform for discussing the biological and Clinical Significance of molecular variants in cancer. To enable users to make complex connections between CIViC information, we developed the CIViC Model Context Protocol (MCP) server, which allows large language models (LLMs) to directly interface with the CIViC API through natural language, facilitating the rapid summarization of expertly curated cancer variant interpretations.

**Availability and implementation:**

The CIViC MCP server is detailed at https://github.com/griffithlab/civic-mcp-server with archived code and evaluation data deposited in Zenodo (DOI: 10.5281/zenodo.17344050). The repository is a fork of https://github.com/QuentinCody/civic-mcp-server (QuentinCody 2025) and includes instructions for accessing the server through the Claude desktop app and hosting it locally with GPT-5. We also provide a Python script for directly querying the MCP server.

**Supplemental information:**

Supplementary data are available at *Bioinformatics* online.

## 1. Introduction

The identification of molecular variants drives precision oncology, informing clinical decision-making and treatment strategies. To give just one illustrative example, ALK fusion variants occur in fewer than 5% of non-small cell lung cancers but can be targeted by kinase inhibitors, such as crizotinib, for significant improvements in patient outcomes (Kwak et al. 2010). The complex mutational landscape of cancers makes the development of molecular variant knowledgebases critical to the goal of enabling the use of genomic information in precision oncology. To address this, the Clinical Interpretation of Variants in Cancer (CIViC) knowledgebase provides a community-driven, open-source platform for assessing the importance of molecular variants in cancer (available at civicdb.org) (Griffith et al. 2017; Danos et al. 2019; Krysiak et al. 2022). CIViC curates evidence items from peer reviewed primary literature as its fundamental unit of information. Each item is assigned an Evidence Type that reflects the variant’s clinical or biological effect, including diagnostic, predictive, prognostic, predisposing, oncogenic, or functional. Each Evidence Type has a corresponding Significance. For example, predictive evidence can indicate whether a tumor is sensitive or resistant to a specific therapy. CIViC assertions are created to summarize collections of evidence items within a specific cancer context, adhering to recognized guidelines (Li et al. 2017; Horak et al. 2022).

**Figure 1.**
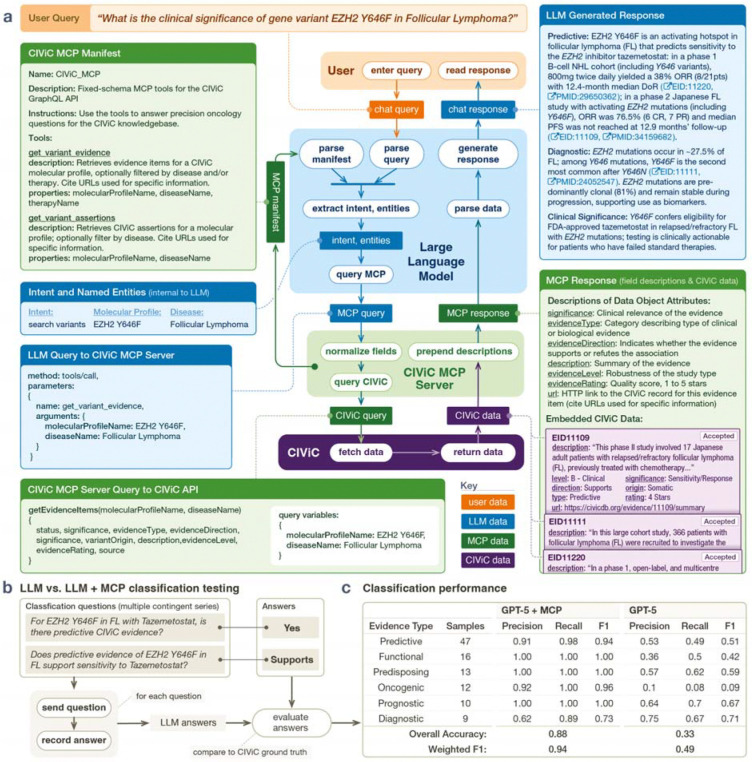
CIViC MCP workflow and evaluation. **(a) Workflow.** A user asks, “What is the clinical significance of EZH2 Y646F in Follicular Lymphoma?” The LLM: (1) reads the MCP manifest and tool descriptions, (2) extracts intent and named entities (molecular profile, disease, therapy), (3) selects the appropriate MCP tool, (4) fills parameters and issues a fixed-schema GraphQL query via the CIViC MCP server, (5) the MCP server queries the CIViC API and returns structured data, (6) the MCP server enriches records with concise field descriptions, (7) the LLM composes a summary. **(b) Evaluation setup.** We formed question/answer pairs from 100 CIViC triplets (molecular profile, disease, therapy). For each triplet, the model first identified which Evidence Types have CIViC evidence and then assigned the corresponding Significance label (supported, not supported, or lacking evidence). These are presented as sets of contingent questions. **(c) Classification performance.** CIViC MCP enhances precision, recall, and F1.

LLMs provide a natural-language interface to CIViC, enabling users to discover, aggregate, and summarize curated oncology knowledge without navigating to multiple pages. They excel at transforming unstructured biomedical text into structured outputs through few- or zero-shot learning and entity normalization. However, general-purpose LLMs cannot guarantee coverage of specialized, rapidly updated resources like CIViC, and they may misinterpret CIViC’s data model, fabricating details or citations when asked for fine-grained clinical information (Bhattacharyya et al.). Without API integration, LLMs fall back on unstructured web browsing, where discoverability is gated by the performance of their search mechanism. Because CIViC’s curation is intentionally granular, captured in individual evidence items for transparency and provenance, many users may benefit from a conversational layer that retrieves the right items and composes succinct, well-cited answers. To provide this layer, we developed a Model Context Protocol (MCP) server that allows LLMs to query CIViC information seamlessly and provide detailed answers to users (Figure 1). MCP servers expose external tools and data sources through a simple, standardized interface, enabling LLMs to issue user-guided queries and receive structured results. In biomedicine, MCP servers are increasingly wrapping domain APIs, databases, and literature search endpoints (Kuehl et al. 2025). The CIViC MCP server benefits both new and experienced users by supporting complex, reproducible queries and rapid, citation-rich summaries.

The CIViC MCP server leverages CIViC’s public GraphQL API to facilitate user-guided queries. The GraphQL schema makes the API straightforward for LLMs to use as objects are explicitly linked (e.g., Evidence Item → Molecular Profile → Variant), letting models see how to move from a clinical question to the exact fields it needs. Through introspection, the LLM reads the schema, discovers types, fields, arguments, and deprecations, and then composes precise queries that fetch only the necessary data. The CIViC knowledge model is highly domain-specific, including complex concepts such as “Molecular Profiles” and “Assertions,” which have precise meanings specific to the platform and may be misinterpreted by an LLM (Krysiak et al. 2022). Despite GraphQL introspection, early tests revealed that Claude Sonnet 4 frequently mis-specified types and arguments, leading to invalid or overly broad queries. While introspection did improve reliability on later queries, where the LLM could contextualize GraphQL error messages against the schema and refine its requests, it did not close the gap relative to predefined tools. We circumvent this challenge in the current implementation of the CIViC MCP server by predefining the GraphQL queries, rather than allowing an LLM to write them to ensure reliability.

## 2. CIViC MCP Server

The CIViC MCP server is hosted on Cloudflare with public access (https://civic-mcp-server.larscivic.workers.dev/mcp). It exposes two tools with predefined GraphQL queries that retrieve CIViC Evidence Items or Assertions for a specified Molecular Profile name (required). Cancer type and therapy are optional filters: when provided, they further restrict results to the most relevant subset; when omitted, the server returns items associated with the given molecular profile across cancer types and therapies. Both tools respond with the URL of each evidence item/assertion when called, making it easy to verify any claims made by the LLM containing specific CIViC information. Each evidence item is also associated with a PubMed ID, which can be further inspected for validation. The JSON object returned by the MCP server also includes a definition of each CIViC field to facilitate LLM summarization.

To handle spelling differences and alternate names, the MCP server normalizes user inputs (molecular profiles, cancer types, therapies) to the closest CIViC preferred label. The preferred label is the canonical, API-recognized name used for exact matching. The MCP server scores candidates with Dice’s coefficient over bigrams and selects the highest-scoring alias. We chose the Dice-Sørensen coefficient because it is fast and memory light, making it a good fit for Cloudflare Workers despite more complex matchers offering higher accuracy. The VICC Gene Normalization Service identifies gene aliases (Kuzma, Stevenson and Wagner 2025). Variant aliases are extracted from the list of curated aliases in CIViC. Cancer types and therapy aliases are extracted from the Disease Ontology and the NCI Thesaurus, respectively (Sioutos et al. 2007; Schriml et al. 2012).

### 2.1 Example Use Case

A user asks, “What is the clinical significance of EZH2 Y646F in follicular lymphoma?” The LLM extracts the entities (molecular profile, disease, therapy if present) and normalizes them to CIViC preferred labels (Figure 1). It selects the get_variant_evidence tool and supplies the necessary parameters. The MCP server then issues a predefined GraphQL query to the CIViC API and returns structured records: Evidence Items with fields such as evidence type, significance, direction, disease, therapy, description, evidence level, rating, and URLs. Using this MCP response, the LLM composes an information-rich answer with links to specific CIViC entries and underlying publications to support the response. In this case, it summarizes the clinical relevance, including describing several clinical trials, for EZH2 Y646F’s role as an activating mutation in follicular lymphoma (FL), a potential diagnostic biomarker for FL, and a biomarker of sensitivity to tazemetostat.

## 3. Evaluation

We selected a controlled classification task: given a CIViC triplet (molecular profile, cancer type, therapy), the LLM must determine which CIViC Evidence Types that triplet has evidence for. Then, for each applicable type, it must assign the corresponding Significance. To assess how effectively LLMs can retrieve and interpret current CIViC information through the MCP server, we compared GPT-5’s performance with and without the MCP server on the same set of 100 randomly selected triplets from CIViC evidence items. We used a two-step prompt at temperature = 1 (Supplementary Prompts 1–4). GPT-5 was first prompted to identify which Evidence Types were relevant to each triplet with a series of yes/no questions. Among the Evidence Types identified as relevant, we prompted GPT-5 to identify each corresponding Significance as being supported, not supported, or lacking evidence. Each triplet could have multiple associated Evidence Types and Significances. Performance is reported at the level of each Evidence Type, with group-level scores calculated by micro-averaging across all associated Significance labels (Figure 1b). We excluded the common “No Evidence” label to avoid inflating global metrics and obscuring errors. GPT-5 + MCP achieved a weighted F1-score of 94.2%. Without the MCP server, GPT-5’s performance dropped to an overall F1-score of 49.4%. Oncogenic evidence proved most challenging for GPT-5, with an F1-score of just 9%, likely reflecting the limited number of such examples in its pretraining data.

We also evaluated ChatGPT-5’s agent mode, which crawls websites by simulating human inputs, on ten randomly selected CIViC triplets. Agent mode achieved perfect accuracy, correctly identifying the Clinical Significance of all ten triplets, whereas GPT-5 alone succeeded on only four (Supplementary Table 3). We then compared response times between the MCP server and agent mode. For 100 triplets, the MCP server had a mean latency of 3.51 seconds (95% CI: 3.34–3.67). In contrast, agent mode required substantially longer, with a mean latency of 306 seconds (95% CI: 132–480) across ten triplets.

## 4. Conclusion

We demonstrate that MCP servers provide standardized, LLM-friendly gateways to curated biomedical databases, enabling precise retrieval and structured summarization. Connecting CIViC to LLMs through MCP improves the accuracy of extracting Clinical Significance in variant-disease-therapy contexts compared with using an LLM’s web client, and it reduces latency relative to agent-mode browsing while maintaining fidelity. By offering structured, real-time access to curated oncology knowledge, the CIViC MCP server mitigates gaps in pretraining coverage, reduces hallucination risk, and yields reproducible, citation-rich outputs. MCP-mediated access both improves accuracy and shortens time to answer. Future work will develop constrained query-composition methods that complement predefined tools, enabling the safe execution of LLM-written queries to address unanticipated user questions. The CIViC MCP server can also be extended to integrate with complementary knowledgebases (e.g., DGIdb, ClinVar, OncoKB), enabling unified multi-database retrieval and cross-knowledge summarization through a single conversational interface.

## Supplementary Material

Supplement 1

## References

[R1] Bhattacharyya M, Miller VM, Bhattacharyya D High Rates of Fabricated and Inaccurate References in ChatGPT-Generated Medical Content. Cureus 15:e39238. 37337480 10.7759/cureus.39238 PMC10277170

[R2] Cannon M, Stevenson J, Stahl K DGIdb 5.0: rebuilding the drug-gene interaction database for precision medicine and drug discovery platforms. Nucleic Acids Res 2024;52:D1227–35. 37953380 10.1093/nar/gkad1040 PMC10767982

[R3] Danos AM, Krysiak K, Barnell EK Standard operating procedure for curation and clinical interpretation of variants in cancer. Genome Med 2019;11:76. 31779674 10.1186/s13073-019-0687-x PMC6883603

[R4] Griffith M, Spies NC, Krysiak K CIViC is a community knowledgebase for expert crowdsourcing the clinical interpretation of variants in cancer. Nat Genet 2017;49:170–4. 28138153 10.1038/ng.3774 PMC5367263

[R5] Horak P, Griffith M, Danos AM Standards for the classification of pathogenicity of somatic variants in cancer (oncogenicity): Joint recommendations of Clinical Genome Resource (ClinGen), Cancer Genomics Consortium (CGC), and Variant Interpretation for Cancer Consortium (VICC). Genet Med Off J Am Coll Med Genet 2022;24:986–98. 10.1016/j.gim.2022.01.001 PMC9081216 35101336

[R6] Krysiak K, Danos AM, Saliba J CIViCdb 2022: evolution of an open-access cancer variant interpretation knowledgebase. Nucleic Acids Res 2022;51:D1230–41. 10.1093/nar/gkac979 PMC9825608 36373660

[R7] Kuehl M, Schaub DP, Carli F Community-based biomedical context to unlock agentic systems. 2025:2025.07.21.665729.

[R8] Kuzma K, Stevenson J, Wagner A. VICC Gene Normalization Service. 2025, DOI: 10.5281/zenodo.16272753.

[R9] Kwak EL, Bang Y-J, Camidge DR Anaplastic lymphoma kinase inhibition in non-small-cell lung cancer. N Engl J Med 2010;363:1693–703. 20979469 10.1056/NEJMoa1006448 PMC3014291

[R10] Li MM, Datto M, Duncavage EJ Standards and Guidelines for the Interpretation and Reporting of Sequence Variants in Cancer. J Mol Diagn JMD 2017;19:4–23. 27993330 10.1016/j.jmoldx.2016.10.002 PMC5707196

[R11] QuentinCody. QuentinCody/civic-mcp-server. 2025.

[R12] Schriml LM, Arze C, Nadendla S Disease Ontology: a backbone for disease semantic integration. Nucleic Acids Res 2012;40:D940–6. 22080554 10.1093/nar/gkr972 PMC3245088

[R13] Sioutos N, de Coronado S, Haber MW NCI Thesaurus: a semantic model integrating cancer-related clinical and molecular information. J Biomed Inform 2007;40:30–43. 16697710 10.1016/j.jbi.2006.02.013

