# Supplementary material for: CIViC MCP: Integrating Large Language Models with the Clinical Interpretations of Variants in Cancer
Source: bioRxiv. 2025 Oct 16:2025.10.13.682185. Preprint. [Version 1] doi: 10.1101/2025.10.13.682185 (PMC12632937; doi:10.1101/2025.10.13.682185)
Supplement: Supplement 1 [file media-1.docx]

**Supplementary Data**

**
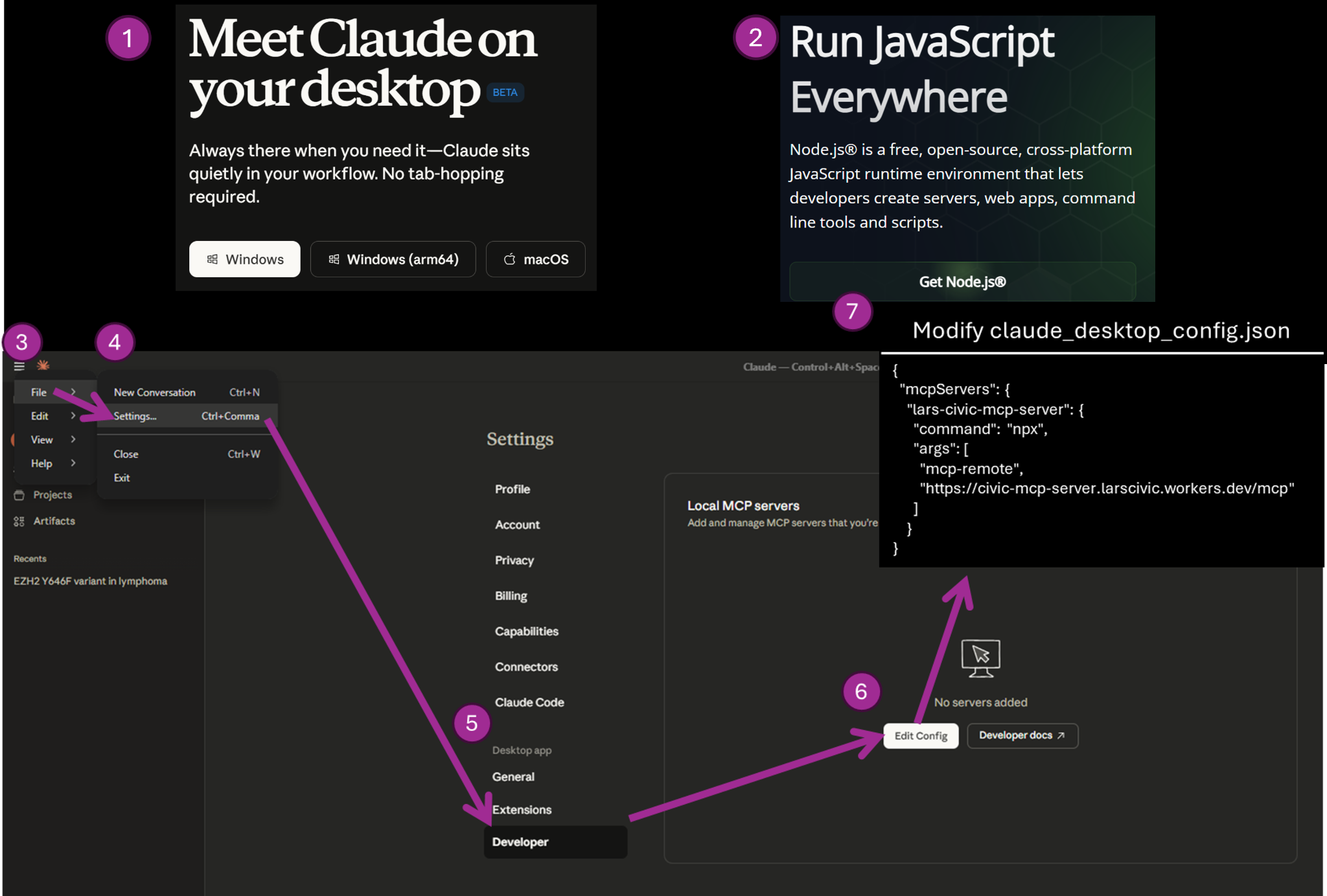
 Supplementary Figure 1.** To interface with the CIViC MCP server through Claude Desktop, first install the application at <https://claude.ai/download>. Then install Node.js (LTS) from [https://nodejs.org](https://nodejs.org/en). After modifying claude_desktop_config.json, restart Claude Desktop. Claude will now have access to the CIViC MCP server during chats.

**Prompt 1. GPT-5 + MCP, Get Evidence Type**

System: You are an expert biomedical annotator trained to assess variant evidence from the Clinical Interpretations of Variants in Cancer (CIViC) knowledgebase given a specific gene variant + cancer + therapy. Use the tools to answer oncology questions for the CIViC knowledgebase.

User: Does the CIViC evidence for this combination of gene variant: {gene_variant}, cancer type: {cancer_type}, and therapy: {therapy} pertain to the {evidence_type} evidence type?

A. Yes

B. No

C. Unsure

Answer with only the letter corresponding to your choice.

**Prompt 2. GPT-5, Get Evidence Type**

System: You are an expert biomedical annotator trained to assess variant evidence from the CIViC knowledgebase given a specific gene variant + cancer + therapy.

Definition of Evidence Types

• Diagnostic – Evidence pertains to a variant’s impact on patient diagnosis (cancer subtype).

• Predictive – Evidence pertains to a variant’s effect on therapeutic response.

• Prognostic – Evidence pertains to a variant’s impact on disease progression, severity, or patient survival.

• Predisposing – Evidence pertains to a germline molecular profile’s role in conferring susceptibility to disease (including pathogenicity evaluations).

• Oncogenic – Evidence pertains to a somatic variant’s involvement in tumor pathogenesis as described by the Hallmarks of Cancer.

• Functional – Evidence pertains to a variant that alters biological function from the reference state.

User: Does the CIViC evidence for this combination of gene variant: {gene_variant}, cancer type: {cancer_type}, and therapy: {therapy} pertain to the{evidence_type} evidence type?

A. Yes

B. No

C. Unsure

Answer with only the letter corresponding to your choice.

**Prompt 3. GPT-5 + MCP, Get Evidence Significance**

System: You are an expert biomedical annotator trained to assess variant evidence from the Clinical Interpretations of Variants in Cancer (CIViC) knowledgebase given a specific gene variant + cancer + therapy. Use the tools to answer oncology questions for the CIViC knowledgebase.

User: Does the {evidence_type} evidence for this combination of gene variant: {gene_variant}, cancer type: {cancer_type}, and therapy: {therapy} support the significance: {significance} in CIViC?

A. Supports

B. Does Not Support

C. No Evidence

Choose all that apply. If both supporting and not supporting evidence exist for the significance {significance}, return A,B. Return C if there is no evidence with this significance. Return B only if there is evidence with this significance

**Prompt 4. GPT-5 + MCP, Get Evidence Significance**

System: You are an expert biomedical annotator trained to assess variant evidence from the CIViC knowledgebase given a specific gene variant + cancer + therapy.

Definition of Evidence Types

• Diagnostic – Evidence pertains to a variant’s impact on patient diagnosis (cancer subtype).

• Predictive – Evidence pertains to a variant’s effect on therapeutic response.

• Prognostic – Evidence pertains to a variant’s impact on disease progression, severity, or patient survival.

• Predisposing – Evidence pertains to a germline molecular profile’s role in conferring susceptibility to disease (including pathogenicity evaluations).

• Oncogenic – Evidence pertains to a somatic variant’s involvement in tumor pathogenesis as described by the Hallmarks of Cancer.

• Functional – Evidence pertains to a variant that alters biological function from the reference state.

User: Does the {evidence_type} evidence for this combination of gene variant: {gene_variant}, cancer type: {cancer_type}, and therapy: {therapy} support the significance: {significance} in CIViC?

A. Supports

B. Does Not Support

C. No Evidence

Choose all that apply. If both supporting and not supporting evidence exist for the significance {significance}, return A,B. Return C if there is no evidence with this significance. Return B only if there is evidence with this significance

**Table 1. GPT-5 + MCP Significance Results (Excluding “No Evidence”)**

| **Evidence Type** | **Significance** | **N** | **Precision** | **Recall** | **F1** |
| --- | --- | --- | --- | --- | --- |
| predictive | sensitivity_response | 29 | 0.908 | 0.966 | 0.931 |
| predictive | resistance | 16 | 0.958 | 1.000 | 0.969 |
| predisposing | predisposition | 13 | 1.000 | 1.000 | 1.000 |
| oncogenic | oncogenicity | 12 | 0.924 | 1.000 | 0.960 |
| diagnostic | positive | 8 | 0.800 | 1.000 | 0.889 |
| functional | loss_of_function | 6 | 1.000 | 1.000 | 1.000 |
| prognostic | better_outcome | 5 | 1.000 | 1.000 | 1.000 |
| functional | gain_of_function | 5 | 1.000 | 1.000 | 1.000 |
| functional | dominant_negative | 5 | 1.000 | 1.000 | 1.000 |
| predictive | reduced_sensitivity | 4 | 1.000 | 1.000 | 1.000 |
| functional | unaltered_function | 2 | 1.000 | 1.000 | 1.000 |
| diagnostic | negative | 1 | 1.000 | 1.000 | 1.000 |
| functional | neomorphic | 1 | 0.000 | 0.000 | 0.000 |
| predisposing | protectiveness | 0 | - | - | - |
| oncogenic | protectiveness | 0 | - | - | - |
| predictive | adverse_response | 0 | - | - | - |
| functional | uncertain_significance | 0 | - | - | - |

**Table 2. GPT-5 Significance Results (Excluding “No Evidence”)**

| **Evidence Type** | **Significance** | **N** | **Precision** | **Recall** | **F1** |
| --- | --- | --- | --- | --- | --- |
| predictive | sensitivity_response | 29 | 0.882 | 0.448 | 0.591 |
| predictive | resistance | 16 | 0.577 | 0.500 | 0.536 |
| predisposing | predisposition | 13 | 0.571 | 0.615 | 0.593 |
| oncogenic | oncogenicity | 12 | 0.104 | 0.083 | 0.093 |
| diagnostic | positive | 8 | 0.750 | 0.750 | 0.750 |
| functional | loss_of_function | 6 | 0.375 | 0.500 | 0.429 |
| prognostic | better_outcome | 5 | 0.600 | 0.600 | 0.600 |
| functional | gain_of_function | 5 | 0.667 | 0.800 | 0.727 |
| functional | dominant_negative | 5 | 0.600 | 0.600 | 0.600 |
| predictive | reduced_sensitivity | 4 | 0.375 | 0.500 | 0.429 |
| functional | unaltered_function | 2 | 0.143 | 1.000 | 0.250 |
| diagnostic | negative | 1 | 0.000 | 0.000 | 0.000 |
| functional | neomorphic | 1 | 0.000 | 0.000 | 0.000 |
| predisposing | protectiveness | 0 | 0.000 | 0.000 | 0.000 |
| oncogenic | protectiveness | 0 | - | - | - |
| predictive | adverse_response | 0 | 0.000 | 0.000 | 0.000 |
| functional | uncertain_significance | 0 | 0.000 | 0.000 | 0.000 |

**Prompt 5. ChatGPT-5 Agent Mode**

User: What is the clinical significance of {gene_variant} in {cancer_type} with {therapy} in CIViC?

Author Note: Fields and filler words (‘in’, ‘with’) are omitted if the tuple value is empty.

**Table 3. ChatGPT-5 Agent Mode Reported Run Times (8/20/2025)**

| **Gene Variant** | **Cancer Type** | **Therapy** | **GPT-5 Correctness** | **Agent Mode Correctness** | **Chat Replay** | **Time (Minutes)** |
| --- | --- | --- | --- | --- | --- | --- |
| ABL1 E453V | Chronic Myeloid Leukemia | Dasatinib | Missed supports sensitivity. | Correct | <https://chatgpt.com/share/68a617e4-efbc-8002-80f1-afad434a2a88> | 2 |
| PIK3R2 G373R | - | - | Missed supports gain of function. | Correct | <https://chatgpt.com/share/68a62229-ab28-8002-9613-a63efdc1afcb> | 5 |
| VHL H115R (c.344A>G) | Von Hippel-Lindau Disease | - | Missed supports predisposition. | Correct | <https://chatgpt.com/share/68a62254-87dc-8002-b936-14ea209fdf8e> | 12 |
| VEGFA VEGF165b Overexpression | Colorectal Cancer | Bevacizumab | Incorrectly said supports resistance. | Correct | <https://chatgpt.com/share/68a62265-4ba8-8002-8f25-1eca4b9603f3> | 2 |
| BCR-ABL1 Fusion AND ABL1 F359I | Chronic Myeloid Leukemia | Imatinib Mesylate | Incorrectly said supports reduced sensitivity. | Correct | <https://chatgpt.com/share/68a62279-82d8-8002-a85c-0827204a3c27> | 6 |
| BRAF V600E | Melanoma | Trametinib | Correct | Correct | <https://chatgpt.com/share/68a623cd-293c-8002-9e51-93ba1a3d5169> | 6 |
| VHL W117fs (c.349dup) | Von Hippel-Lindau Disease | - | Correct | Correct | <https://chatgpt.com/share/68a62472-c7d8-8002-8bf5-50128a30fa2c> | 2 |
| TPM3-NTRK1 Fusion | Lung Carcinoma | Larotrectinib | Correct | Correct | <https://chatgpt.com/share/68a62780-2b20-8002-8dff-9721d666e35b> | 12 |
| VHL P146fs (c.437del) | Von Hippel-Lindau Disease | - | Incorrectly said supports predisposition. | Correct | <https://chatgpt.com/share/68a6281e-cacc-8002-a92a-1d51a5e21397> | 1 |
| TP53 Mutation | Lung Adenocarcinoma | - | Correct | Correct | <https://chatgpt.com/share/68a6290e-3714-8002-9e09-121d37ce59a8> | 3 |

GPT-5 Correctness indicates if the GPT-5 without MCP identified the correct significance for the ten randomly selected triplets. We then compare this to the GPT-5 agent response (Prompt 5) to see if the correct significance was mentioned. The ChatGPT-5 agent mode correctly identified the significance of all triplets.

Average time: 5.1 minutes with a 95% confidence interval of 2.2-8.0 minutes.

Chat contents are also available at<https://github.com/griffithlab/civic-mcp-server/tree/defined_CIViC_tools/eval_QA_experiment/agent_mode>
